# Dual-Energy CT Material Decomposition: The Value in the Detection of Lymph Node Metastasis from Breast Cancer

**DOI:** 10.3390/diagnostics14050466

**Published:** 2024-02-21

**Authors:** Ibrahim Yel, Tommaso D’Angelo, Leon D. Gruenewald, Vitali Koch, Rejane Golbach, Scherwin Mahmoudi, Giorgio Ascenti, Alfredo Blandino, Thomas J. Vogl, Christian Booz, Giuseppe M. Bucolo

**Affiliations:** 1 Division of Experimental Imaging, University Hospital, Goethe University Frankfurt, 60590 Frankfurt am Main, Germany; gruenewald.leon@me.com (L.D.G.); boozchristian@gmail.com (C.B.); giuseppebucolo94@gmail.com (G.M.B.); 2 Clinic for Radiology and Nuclear Medicine, University Hospital, Goethe University Frankfurt, 60590 Frankfurt am Main, Germany; vitali-koch@gmx.de (V.K.);; 3 Department of Biomedical Sciences and Morphological and Functional Imaging, University of Messina, 98122 Messina, Italygiorgio.ascenti@unime.it (G.A.); ablandino@unime.it (A.B.); 4 Department of Radiology and Nuclear Medicine, Erasmus MC, 3015 GD Rotterdam, The Netherlands; 5 Institute of Biostatistics and Mathematical Modelling, University Hospital Frankfurt, 60596 Frankfurt am Main, Germany; golbach@med.uni-frankfurt.de

**Keywords:** fat fraction, dual energy, breast cancer, lymphatic metastases

## Abstract

Purpose: To evaluate the diagnostic performance of a dual-energy computed tomography (DECT)-based material decomposition algorithm for iodine quantification and fat fraction analysis to detect lymph node metastases in breast cancer patients. Materials and Methods: 30 female patients (mean age, 63.12 ± 14.2 years) diagnosed with breast cancer who underwent pre-operative chest DECT were included. To establish a reference standard, the study correlated histologic repots after lymphadenectomy or confirming metastasis in previous/follow-up examinations. Iodine concentration and fat fraction were determined through region-of-interest measurements on venous DECT iodine maps. Receiver operating characteristic curve analysis was conducted to identify the optimal threshold for differentiating between metastatic and non-metastatic lymph nodes. Results: A total of 168 lymph nodes were evaluated, divided into axillary (metastatic: 46, normal: 101) and intramammary (metastatic: 10, normal: 11). DECT-based fat fraction values exhibited significant differences between metastatic (9.56 ± 6.20%) and non-metastatic lymph nodes (41.52 ± 19.97%) (*p* < 0.0001). Absolute iodine concentrations showed no significant differences (2.25 ± 0.97 mg/mL vs. 2.08 ± 0.97 mg/mL) (*p* = 0.7999). The optimal fat fraction threshold for diagnosing metastatic lymph nodes was determined to be 17.75%, offering a sensitivity of 98% and a specificity of 94%. Conclusions: DECT fat fraction analysis emerges as a promising method for identifying metastatic lymph nodes, overcoming the morpho-volumetric limitations of conventional CT regarding lymph node assessment. This innovative approach holds potential for improving pre-operative lymph node evaluation in breast cancer patients, offering enhanced diagnostic accuracy.

## 1. Introduction

Breast cancer is a significant health concern, representing the primary malignant neoplasm in women and accounting for the leading cause of cancer-related mortality within the female population [1,2,3]. The presence of lymph node involvement plays a critical role as a prognostic factor, with a substantial impact on 5-year survival rates, resulting in a decrease of up to 40% when lymph node metastases are present [4]. Consequently, European guidelines recommend imaging of the axillary region at the time of breast cancer diagnosis to appropriately determine the course of (axillary) treatment based on imaging results [5,6].

The pre-surgical evaluation of lymph node status holds immense importance, as it substantially influences the selection of appropriate post-surgical interventions, such as chemotherapy or radiotherapy [7]. The performance of radical mastectomy necessitated complete axillary lymph node excision to accurately identify metastases. However, contemporary medical practices have leaned towards a more conservative approach to minimize immediate and long-term complications, including pain and lymphedema. This approach typically involves the excision of several “random” lymph nodes, typically around four, during the initial surgery. This is coupled with a sentinel lymph node biopsy, which aids in identifying primary lymphatic drainage pathways and isolating the most likely metastatic lymph nodes for subsequent biopsy [7]. In cases where lymph node metastases are confirmed, a complete axillary lymph node dissection is usually recommended, although axillary radiation therapy presents itself as a viable alternative. Sentinel lymph node biopsy stands as the surgical standard for patients with clinically and radiologically negative lymph nodes.

In the realm of pre-operative breast cancer management, computed tomography (CT) imaging plays a pivotal role in tumor staging, assessing disease extent, and determining appropriate surgical and medical treatment options. However, conventional CT only provides morpho-volumetric information to differentiate between healthy and metastatic lymph nodes.

Typically, normal lymph nodes are characterized by their oval shape, homogeneity, a short-axis measurement of less than 1 cm, and the presence of an adipose hilus. In contrast, metastatic lymph nodes may display increased size, irregular morphology, inhomogeneous density, and the potential presence of a colliquated core. Consequently, the diagnostic accuracy of morphological imaging has limitations, as neoplastic infiltration can occur within small-diameter lymph nodes as well [8,9].

Moreover, it is essential to emphasize the growing significance of non-invasive diagnostic tools, such as DECT, to complement and enhance traditional diagnostic methods. The field of medical imaging continues to evolve, with emerging technologies offering new avenues for improving patient care and outcomes. DECT represents a pioneering approach that leverages material decomposition algorithms to provide a more comprehensive understanding of tissue composition. By analyzing the differential absorption characteristics of X-ray beams at varying energies, DECT enables precise quantification of iodine concentration and fat fraction. This innovative approach not only addresses the limitations of conventional CT but also offers a potential breakthrough in the field of breast cancer diagnosis.

Hence, the primary objective of this study is to evaluate the effectiveness of the Dual-Energy Computed Tomography (DECT) material decomposition algorithm in differentiating between metastatic and non-metastatic lymph nodes in patients diagnosed with breast cancer. This innovative approach aims to improve the accuracy of lymph node assessment, providing valuable insights for more precise treatment decisions.

## 2. Materials and Methods

### 2.1. Ethical Considerations

This retrospective study strictly adhered to ethical guidelines and received approval from our institutional review board. Informed consent requirements were waived due to the retrospective nature of the study.

### 2.2. Study Population

The study involved a comprehensive review of our institutional databases to identify adult patients who had undergone surgery involving lymph node excision and had pre-operative dual-energy chest CT scans conducted between January 2022 and March 2023. We specifically focused on lymph node metastasis in the first three levels of lymphatic drainage of the breast, to align with clinically relevant parameters for breast cancer lymphatic spread.

**Figure 1:**
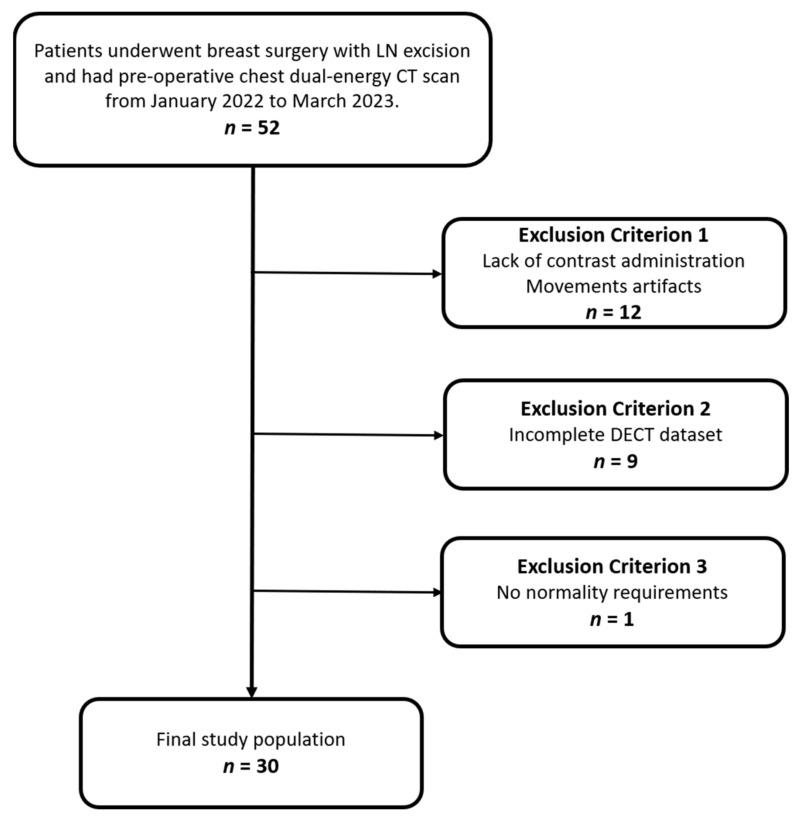
Flowchart of patient inclusion and exclusion criteria.

Patients were excluded if they did not receive a contrast medium or if their DECT datasets were incomplete. Additionally, individuals who had their CT scans more than a month before surgery were also excluded from the study. A visual representation of the patient selection process is illustrated in Figure 1.

### 2.3. DECT Imaging Technique

All CT scans were performed using the same third-generation dual-source DECT scanner (SOMATOM Force, Siemens Healthineers, Forchheim, Germany). Image acquisition was performed in the craniocaudal direction during the inspiratory breath-hold maneuver.

The study protocol consisted of acquiring the venous phase in dual-energy mode, which was initiated 80 s after the administration of the contrast medium. The contrast agent Iomeprol (Imeron 350, Bracco Imaging, Konstanz/Germany) was intravenously administered at a dose of 1.2 mL/kg of body weight through a peripheral forearm vein. The contrast media administration occurred at a rate of 2–3 mL/s, with a maximum limit of 120 mL, followed by an 80 mL saline flush.

DECT acquisition settings were precisely configured as follows: tube A: 100 kV, 190 mAs; tube B: 150 kV, 95 mAs; with the additional use of a tin filter (Selective Photon Shield II, Siemens Healthineers). The rotation time and collimation were set at 0.5 s and 2 × 192 × 0.6 mm, respectively. The protocol utilized automatic attenuation-based tube current modulation (CARE Dose 4D; Siemens Healthineers). All images were reconstructed with a section thickness of 3.0 mm and an increment of 2.0 using a soft tissue convolution kernel (Qr40; Siemens Healthineers) as well as a bone kernel (Q69F; Siemens Healthineers).

### 2.4. DECT Mapping and Uptake Measurements

Subsequently, DECT data were systematically transferred to a dedicated post-processing workstation (syngo.via version VA30, Siemens Healthineers), and iodine maps were calculated using the Liver VNC algorithm (Siemens Healthineers).

The liver VNC application enables the visualization of iodine (contrast agent) concentration by isolating the iodine content from the Hounsfield unit value of each voxel. It then presents the pure iodine map as a colored overlay on the grayscale image. Circular region-of-interest (ROI) measurements were conducted by two highly experienced radiologists with five and ten years of expertise. These measurements were performed on lymph nodes with a minimum measurable area of 0.2 cm^2^, with particular care taken to avoid the fatty hilus region. For lymph nodes evaluable across multiple CT slices, we averaged the ROI measurements from all visible slices to ensure a thorough assessment of each lymph node’s characteristics.

**Figure 2:**
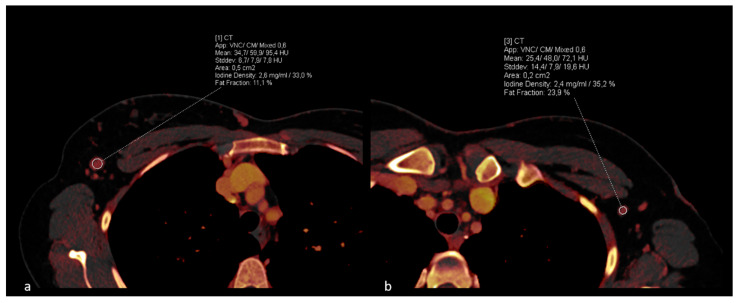
Case example of a patient with histologically confirmed right-sided breast carcinoma. Pre-operative ROI measurements on Dual-Energy CT iodine maps with identification of metastatic lymph nodes in the right axilla (**a**). Contralateral measurement demonstrates a healthy lymph node (**b**).

DECT material decomposition values, including iodine density measured in mg/mL and fat fraction represented as a percentage, were extracted, as depicted in Figure 2. To minimize the influence of patient-specific perfusions on the results, additional measurements of the iodine concentration of the thoracic aorta were performed to achieve data normalization for the iodine density.

Lymph nodes were classified as either metastatic or non-metastatic through consultation with histological reports (and eventually input from the surgeon who performed the excision). Alternatively, classification was made based on pathological morphologic changes compared to a prior or follow-up CT examination. The mean values of the obtained data were utilized for the statistical analysis.

### 2.5. Statistical Analysis

A linear mixed effects regression model was employed for an examination of the differences in fat fraction and iodine density between affected and healthy lymph nodes, accounting for the presence of multiple measurements within patients. The validity of the model assumptions was assessed by scrutinizing the normal distribution of residuals using the Kolmogorov-Smirnov-Lilliefors test.

To meet the model’s requirements regarding the normality of residuals for the fat fraction model, one measurement was selectively excluded from this analysis. The leverage value of this measurement in the initial regression was relatively small (0.142) compared to the mean leverage (0.181).

To ascertain the optimal cutoff value for fat fraction, a comprehensive receiver operating characteristic (ROC) analysis was conducted, utilizing a mixed effects logistic regression model. Mean differences are presented alongside their standard errors. All statistical tests were performed as two-tailed tests, and a significance level of *p* ≤ 0.05 was considered statistically significant. The entirety of the statistical analyses were carried out using R software, version 4.2.1 (R Foundation for Statistical Computing, Vienna, Austria).

## 3. Results

Our final study cohort, characterized by its unbiased selection process without preselection based on BMI, age, or other variables, comprised 30 female patients with an average age of 63.12 ± 14.20 years. Among these patients, 23 were diagnosed with invasive ductal carcinoma, while 7 presented with invasive lobular carcinoma. 18 of the carcinomas were located on the left mamma, while 12 were found on the right side, resulting in a balanced distribution between the sides. For a comprehensive overview of patient characteristics, refer to Table 1.

**Figure 3:**
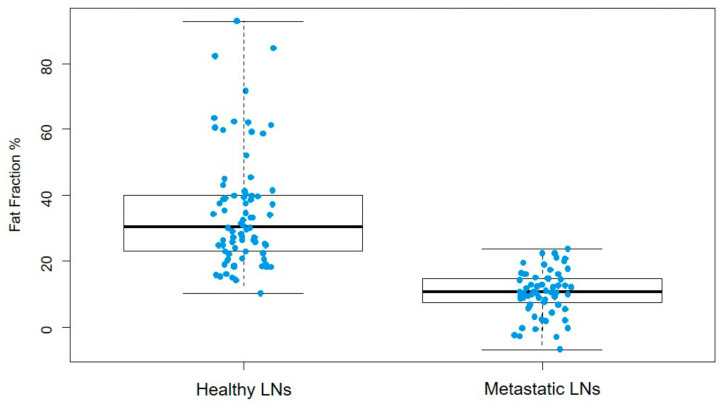
The graph shows the distribution of fat fraction in healthy lymph nodes (LNs) (mean value 41.52 ± 19.97%) and in metastatic ones (9.56 ± 6.2%) (*p* < 0.0001).

A total of 168 lymph nodes underwent evaluation, categorized into two distinct groups: axillary (metastatic: 46, normal: 101), and intramammary (metastatic: 10, normal: 11). The average fat fraction in metastatic lymph nodes was calculated to be 9.56 ± 6.20%, which was significantly lower compared to the fat fraction in healthy lymph nodes, recorded at 41.52 ± 19.97% (*p* < 0.0001) (Figure 3).

**Figure 4:**
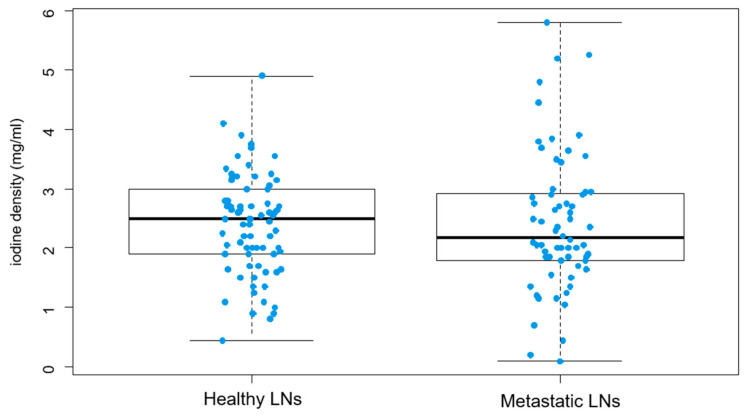
Distribution of iodine density in healthy lymph nodes (LNs) and in metastatic ones is displayed in the graphs. Healthy LNs show a mean iodine density of 2.25 ± 1.14 mg/mL, compared to metastatic LNs (2.08 ± 0.97 mg/mL). The comparison between the two groups shows no statistical difference (*p* = 0.7999).

The mean iodine density value of metastatic lymph nodes was 2.25 ± 1.14 mg/mL and exhibited no significant differences when compared to healthy lymph nodes, which presented with an iodine density of 2.08 ± 0.97 mg/mL (*p* = 0.7999) (Figure 4).

**Figure 5:**
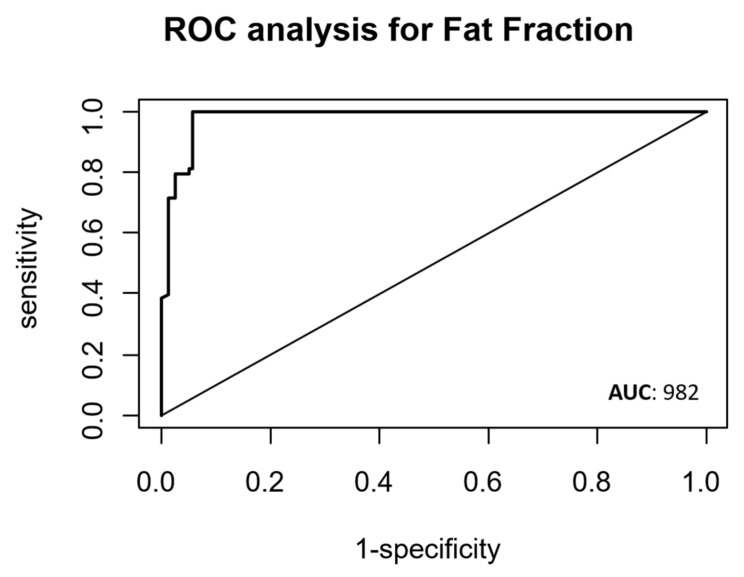
ROC curve shows the diagnostic performance of fat fraction in the assessment of metastatic lymph nodes. The numerical values show the area under the curve (AUC).

Through the ROC analysis, we identified the optimal fat density threshold as 17.75% for distinguishing between metastatic and healthy lymph nodes. This threshold was determined through cross-validation. With this defined threshold, we achieved a sensitivity of 98% (95% CI, 97–100%), a specificity of 94% (95% CI, 68–99%), and an area under the curve (AUC) of 0.982 (Figure 5). All the results are displayed in Table 2.

## 4. Discussion

As the very first study, we evaluated the diagnostic performance of dual-energy computed tomography (DECT)-based material decomposition algorithms in the detection of lymph node metastases among patients with breast cancer. Our findings reveal a substantial disparity in fat fraction between healthy and metastatic lymph nodes, revealing a decline of up to 70%. In contrast, absolute iodine concentration yielded no notable differences between the two groups.

The increasing clinical adoption of dual-energy computed tomography technology in clinical practice has enhanced our comprehension of tissue composition. DECT leverages a material decomposition algorithm to precisely quantify iodine concentration and fat fraction by analyzing the differential absorption characteristics across two X-ray beam energies [10]. Our study underscores the pivotal role of DECT in providing deeper insight into lymph node characteristics, particularly in breast cancer patients.

Typically, healthy lymph nodes are characterized by the presence of fat in the hilum, which can be visualized through various imaging techniques. However, in cases where the fatty hilum is not adequately visible, conventional computed tomography reaches its diagnostic limitations and must rely solely on anatomical shape and size for assessment [8,9,11].

While large, irregularly shaped, and heterogeneous lymph nodes are more likely to be malignant and can be easily identified based on morpho-volumetric criteria, smaller lymph nodes may not be accurately characterized [12,13,14]. Generally, lymph nodes with a short axis diameter ≥ 10 mm or a long axis diameter ≥ 15 mm are considered pathological [15]. Positron emission tomography (PET)-CT proves valuable in such scenarios by integrating oncologic imaging for improved lymph node assessment through functional and metabolic evaluations [16]. Although effective, the availability and cost-efficiency of PET-CT remain inferior to those of conventional CT. In this context, DECT’s potential can be very useful in clinical practice for lymph node characterization, contributing to early and accurate diagnosis and treatment.

DECT’s relevance is growing, driven by ongoing research to validate innovative, novel clinical applications. Algorithms such as virtual monoenergetic imaging, virtual non-contrast, and the use of iodine maps have achieved considerable success in vascular, abdominal, and brain imaging [17,18,19,20,21,22,23,24].

Fat fraction assessment has demonstrated commendable results in quantifying hepatic fat [25,26] and assessing pancreatic adipose degeneration [27]. Furthermore, material decomposition reconstructions showed an important role in the differentiation of various abdominal focal lesions [28,29,30].

Mahmoudi et al. [31] examined iodine density and fat fraction obtained from material decomposition DECT reconstructions to differentiate intrahepatic cholangiocarcinoma from hepatocellular carcinoma. They reported significant differences in iodine concentration and fat fraction, highlighting the potential of DECT material decomposition reconstructions in aiding clinical routines.

Additionally, Martin et al. [32] investigated the value of DECT-derived iodine and fat fraction quantification in distinguishing malignant abdominal lymphoma from lymph node metastases. Their findings demonstrated significant differences between iodine and fat fraction quantifications in characterizing abdominal lymph nodes, offering valuable insights into distinguishing between malignant lymphomas, lymph node metastases, and healthy lymph nodes.

However, it is important to note that material decomposition algorithms have not been previously evaluated for the depiction of lymph node metastases in breast cancer patients.

Our results suggest that fat fraction analysis can significantly contribute to the early identification of lymph node metastases, expediting and simplifying the determination of an appropriate surgical-therapeutic approach. Moreover, the fat fraction exhibits excellent diagnostic accuracy, as indicated by an AUC of 0.982, with a threshold value of 17.75%.

In contrast to our findings, where the decrease in iodine concentration was not significant, previous studies have reported a substantial decrease in iodine concentration within metastatic lymph nodes, compared with healthy lymph nodes in metastatic squamous cell carcinoma of the head-neck [33] and rectal cancer [34]. The divergence between these findings and our own may be attributed to the distinct histological behavior of breast cancer and the relatively small dimension of the lymph nodes analyzed. Consequently, the reduction in fat fraction could potentially indicate an early stage in the metastatic process. Nonetheless, further research efforts are warranted to comprehensively investigate and validate these preliminary results.

In addition, the COVID-19 pandemic introduced new challenges in evaluating axillary lymph nodes, as the COVID-19 vaccination led to lymphadenopathy, particularly in the ipsilateral axilla [35]. This posed a diagnostic dilemma, especially for women with a recent breast cancer diagnosis on the same side as the vaccination. Van Nijnatten et al. [36] discussed the differences in lymphadenopathy characteristics at breast cancer diagnosis versus post-COVID-19 vaccination across various imaging modalities (US, breast MRI, and 18F-FDG PET/CT) and emphasize the importance of documenting clinical information related to vaccination to guide accurate interpretation and treatment decisions. Tissue sampling may be necessary in cases of post-vaccination lymphadenopathy in breast cancer patients [35,36].

Our study does acknowledge certain limitations, including a relatively small population, the inclusion of only surgically treated patients, and the exclusive evaluation of axillary and intramammary lymph nodes. Furthermore, our research was conducted exclusively using one CT scanner, highlighting the need for additional research exploring other dual-energy technologies from different manufacturers. To provide a more focused insight into breast cancer staging, we deliberately limited our analysis to lymph node metastasis within the first three levels of lymphatic drainage, terminating at the lower border of the clavicle. This decision was based on the clinical significance of these regions for breast cancer lymphatic spread. Future studies could benefit from including more lymph node levels to comprehensively assess the diagnostic capabilities of DECT in breast cancer staging. Our study did not specifically analyze differences in radiologic detection of metastatic lymph nodes between invasive ductal and lobular carcinomas due to the limited sample size. Further targeted research is needed to explore these distinctions comprehensively. A limitation of our study is the absence of a standardized process for marking and directly comparing radiologically positive lymph nodes with pathologic outcomes. This approach limited our ability to perform precise, one-to-one correlations, highlighting an area for methodological improvement in future research to enhance the diagnostic accuracy of DECT in lymph node assessment.

## 5. Conclusions

The assessment of fat fraction holds promise as an adjunctive tool for gaining valuable insights into lymph node status in breast cancer, potentially mitigating the morpho-volumetric limitations associated with traditional CT in the depiction of lymph node metastases. Our study has elucidated the prospective utility of fat fraction analysis as a noteworthy diagnostic parameter, showcasing a substantial decline in fat fraction in metastatic lymph nodes, which could serve as an early indicator of malignancy. However, the application of dual-energy CT technology for non-hepatic fat fraction measurement remains an area under scrutiny and has yet to be widely adopted in clinical practice. The full scope of its clinical utility and integration into routine patient care are still being explored. Furthermore, the potential implications of fat fraction analysis extend beyond breast cancer. This innovative approach may find relevance in other oncological areas. Therefore, additional research efforts are imperative to further refine the utility of iodine concentration and fat fraction measurements within a clinical context.

## Figures and Tables

**Table 1:** Patient characteristics.

Characteristic	All Female (*n* = 30)
Mean age ± SD	63.12 ± 14.20 years
Side Carcinoma	
Left	18 (60%)
Right	12 (40%)
Histology	
Invasive ductal carcinoma	23 (76.67%)
Invasive lobular carcinoma	7 (23.33%)
BMI ± SD	26.52 ± 2.56

SD: standard deviation; BMI: body mass index.

**Table 2:** Summary of fat fraction and iodine density in healthy vs. metastatic lymph nodes, indicating differences in mean values, variability, and distributions.

	Fat Fraction		Iodine Density	
	Healthy LNs	Metastatic LNs	Healthy LNs	Metastatic LNs
Sample sizes	138	76	138	76
Lowest Value	10.20	−6.55	0.20	0.10
Highest Value	106.90	23.70	4.90	5.80
Mean Value	41.52	9.56	2.25	2.08
SD	19.97	6.20	0.97	1.14
Median	30.5	10.65	2.5	2.18
IQR	7.55–14.83	22.91–39.95	1.90–2.95	1.8–2.92

LN: lymph node; SD: standard deviation; IQR: interquartile range.

## Data Availability

The data presented in this study are available on request from the corresponding author. The data are not publicly available due to data protection.

## Abbreviations

AUC	Area Under the Curve
DECT	Dual-Energy CT
PET	Positron Emission Tomography
ROC	Receiver Operating Characteristic
ROI	Region of Interest
